# Forgetting in visual working memory: Internal noise explains decay of feature representations

**DOI:** 10.1167/jov.22.8.8

**Published:** 2022-07-15

**Authors:** Crista Kuuramo, Jussi Saarinen, Ilmari Kurki

**Affiliations:** 1 Department of Psychology and Logopedics, University of Helsinki, Helsinki, Finland; 2 Department of Psychology and Logopedics, University of Helsinki, Helsinki, Finland; 3 Department of Psychology and Logopedics, University of Helsinki, Helsinki, Finland

**Keywords:** visual working memory, classification images, signal detection theory

## Abstract

The precision of visual working memory (VWM) representations decreases as time passes. It is often assumed that VWM decay is random and caused by internal noise accumulation. However, forgetting in VWM could occur systematically, such that some features deteriorate more rapidly than others. There exist only a few studies testing these two models of forgetting, with conflicting results. Here, decay of features in VWM was thoroughly tested using signal detection theory methods: psychophysical classification images, internal noise estimation, and receiver operant characteristic (ROC). A modified same–different memory task was employed with two retention times (500 and 4000 ms). Experiment 1 investigated VWM decay using a compound grating memory task, and Experiment 2 tested shape memory using radial frequency patterns. Memory performance dropped some 15% with increasing retention time in both experiments. Interestingly, classification images showed virtually indistinguishable weighting of stimulus features at both retention times, suggesting that VWM decay is not feature specific. Instead, we found a 77% increase in stimulus-independent internal noise at the longer retention time. Finally, the slope of the ROC curve plotted as *z*-scores was shallower at the longer retention time, indicating that the amount of stimulus-independent internal noise increased. Together these findings provide strong support for the idea that VWM decay does not result from a systematic loss of some stimulus features but instead is caused by uniformly increasing random internal noise.

## Introduction

Visual working memory (VWM) is a fundamental human capacity that enables the encoding, storing, and retrieval of information in various cognitive tasks—such as reading, writing, language learning, and measures of fluid intelligence (Engle, 2002; Engle, 2018; Jaeggi, Buschkuehl, Jonides, & Perring, 2008; Luck & Vogel, 2013). Although the very limited capacity of VWM has been thoroughly studied (Cowan, 2001; Luck & Vogel, 1997), there is less research on forgetting in VWM (see, however, Cohen-Dallal, Fradkin, & Pertzov, 2018; Honig, Ma, & Fougnie, 2020; Mercer & Barker, 2020; Pertzov, Manohar, & Husain, 2017; Ricker, Sandry, Vergauwe, & Cowan, 2020; Schneegans & Bays, 2018; Zhang & Luck, 2009). Although most studies on forgetting have demonstrated temporal decay in VWM, some have not (Blake, Cepeda, & Hiris, 1997; Magnussen, Greenlee, Asplund, & Dyrnes, 1990; Magnussen, Greenlee, & Thomas, 1996). Forgetting in VWM might happen only in certain circumstances—for example, when task difficulty is sufficiently high (Skottun, 2004) or when there is interference between memory items (Oberauer & Lin, 2017). To this day, there is no agreement on the mechanisms underlying the decay in VWM; this question has been formally studied in just a few instances (Gao & Bentin, 2011; Gold, Murray, Sekuler, Bennett, & Sekuler, 2005; Harvey, 1986).

In the present study, we used classification images (CIs) and signal detection theory (SDT) to test the models of VWM decay. The basic idea in SDT is to compare human performance in an external noise-limited task with a Bayesian ideal observer that possesses ideal encoding and storage capacity and no internal noise. This allows us to separate two types of inefficiencies in information processing: suboptimal encoding (and/or storage) of information and internal random noise. The first can be thought as systematic, or deterministic, inefficiency, and the latter as random.

In terms of SDT, forgetting due to random decay would manifest as an increase in internal noise as time passes. According to this view, stimulus-independent neural noise, ubiquitous in neural systems, would corrupt memory representations in a random fashion. This means there would be no systematic change in memory representations due to time passing (i.e., no change in which features are sampled). Internal noise would lower the signal-to-noise ratio of memory readout, but not alter the contents of the representation. Several recent studies have implicitly suggested that such a randomly operating mechanism underlies memory decay (Bays, 2014; Bays, 2015; Bays, Catalao, & Husain, 2009; Bays & Husain, 2008; Schurgin, Wixted, & Brady, 2020). Another possibility is that forgetting occurs due to systematic changes in memory representations (Gold et al., 2005); for example, some visual features might decay more rapidly than others and they would be systematically lost. Systematic forgetting could effectively decrease the encoding (sampling) efficiency when the memory representation is recalled and would predict loss in memory performance, even without an increase in internal signal-to-noise ratio.

To our knowledge, only one study (Gold et al., 2005) has explicitly compared these two models of VWM decay using SDT methodology. These authors used bandpass filtered textures, equivalent noise masking, and double-pass methods to directly study whether internal noise level increases or sampling efficiency decreases in the course of forgetting. Their results supported the notion of systematic/deterministic memory decay, as the estimated amount of internal noise did not increase with longer retention times but sampling efficiency decreased. To explain the mechanism of how decreasing sampling efficiency could cause forgetting in VWM, Gold et al. proposed that systematic decay could happen as gradual loss of fine stimulus details. For example, for a stimulus composed of multiple spatial frequencies, forgetting would occur in the highest frequency features first. However, this idea was not directly tested by comparing how forgetting affects each frequency band separately.

### Memory decay tested using classification image method

Here, we used a novel variant of the CI method (Ahumada, 2002; Ahumada & Lovell, 1971; Murray, 2011; Pritchett & Murray, 2015) to investigate memory decay in VWM. The aim of the method is to estimate a CI (i.e., a map of the internal weights) that describes how various stimulus features contribute to the observer's memory-based decisions. This enables testing directly whether or not stored stimulus features systematically change during the memory decay. The CI method has been previously used in perception studies, but here we developed a variant that can be used to probe memory representations in our version of the same–different change detection task that is commonly employed to study VWM.

We collected data from two experiments that targeted different stages of visual processing. In Experiment 1, we used compound gratings that are generally thought to probe the mechanisms of low-level spatial vision. In Experiment 2, we employed radial frequency patterns, which are commonly thought to index mid-level visual mechanisms (see, for example, Loffler, 2015). Both sets of stimuli are difficult to verbalize, promoting purely visual memory strategies. As our stimuli were composed of multiple frequencies components, we could directly test whether forgetting in VWM is a systematic process (i.e., so that the highest frequencies are lost first). Note, however, that this is just one way of describing systematic decay in VWM. Forgetting in VWM could operate by other systematic changes, such as a loss of detail in the periphery of the visual field, which are not explored in this study.

Memory stimuli were composed of 10 Fourier components of spatial frequencies (SFs; Experiment 1) or radial frequencies (RFs; Experiment 2) that had constant amplitudes but varying phases. In other words, the observer's task was to memorize the spatial structure of a compound grating (Experiment 1) or the complex contour shape of an RF pattern (Experiment 2). In order to prevent the observer from learning a single and constant set of memory stimuli, the phase between components was randomized for each trial, thus producing a new grating structure or contour shape to be memorized.

A test stimulus was created from a memory stimulus by adding Gaussian distributed random values (external phase noise) independently to each component's phase in the memory stimulus. On the similar trials, noise was generated from a distribution with a small standard deviation, resulting in little difference between the memory and test stimuli in spatial structure (Experiment 1) or contour shape (Experiment 2). For the different trials, the phase noise emerged from a distribution with a large standard deviation so that the component phase difference (and thus appearance) between the memory and test stimuli varied, on average, considerably. We chose to add external noise also to similar trials, whereas many previous studies have used identical stimuli for change detection tasks. Adding a small amount of external noise is required for the classification image method, because for CI estimation the difference of the component phases must be calculated; if the difference is zero, then the CI is undefined (for details, see Equation 2). Further, noise in similar trials makes the task a genuine signal-in-noise signal detection task; that is, there is a non-zero probability that any stimuli can come from either similar or different distributions. This allows us to define an ideal encoding/retrieval strategy.

**Figure 1.**
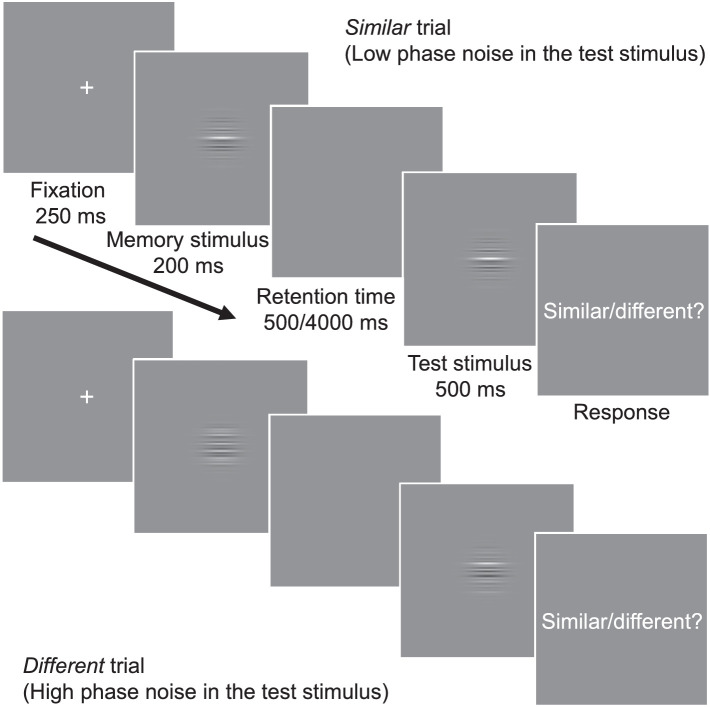
The procedure in Experiment 1. We used a modified same–different memory task with a retention time that was randomly either short (500 ms) or long (4000 ms). The test stimulus was constructed by adding phase noise in the memory stimulus (randomly varying the phase of the grating). In a similar trial, the phase noise added to the test stimulus was low, so that the difference between the memory and test stimuli was small. In a different trial, the noise in test stimulus was larger, thus making the memory and test stimuli appear different.

On each trial, the observer was presented with two stimuli (memory and test stimulus, with a brief retention time between them); an example from Experiment 1 is shown in Figure 1. The observer's task was to respond, using a four-step rating scale, whether the memory and test stimuli appeared similar or different in spatial structure (Experiment 1) or contour shape (Experiment 2). See details of the response options in the Procedure section of Experiment 1.

A CI was then estimated using a regression model that predicted the observer's similar/different responses from the stimulus values that were presented in the experiment on a trial-to-trial basis. The basic idea is that the correlation between phase noise values at certain features and the observer's response indicates how much information from this feature is retained in VWM. To illustrate, consider that the observer retains feature A but not feature B in VWM. The observer should then be more likely to respond “different” to high (absolute) phase noise values of A (as it resembles a different stimulus) and “similar” to low absolute noise values. On the other hand, no value of feature noise B would predict the observer's responses if the representation of feature B is decayed and thus not available in VWM. It is possible to estimate how the observer has used various features for memory-based decisions using a regression model where the observer's responses have been predicted from squared values of phase noise. A specific drop in weighting of some features at longer retention times would then suggest that those features are prone to greater memory decay, thus supporting the notion of systematic/deterministic decay in VWM.

The CI weights thus describe how the retained stimulus features (the frequency components) contribute to decisions. Without memory delay, the weights would index the frequency tuning function of perceptual encoding. Potential change in relative weights describes the frequency tuning function for forgetting. Spatial (Campbell & Robson, 1968; Graham, 1989) and radial (Bell, Badcock, Wilson, & Wilkinson, 2007; Loffler, 2015; Wilkinson, Wilson, & Habak, 1998) frequency components are known to have important role in perceptual encoding, and different components may also carry out different types of physical information: Low SFs convey information from surfaces and gross illumination changes, and high SFs convey contours and texture details (De Valois & De Valois, 1980); low RFs convey gross shape information, and high RFs convey fine details (Wilkinson et al., 1998). Note, however, that the method does not assume that frequency components are stored in WM as independent entities or “items.” Although VWM representation itself may have more abstract rather than feature-based representation, it is still decodable back to stimulus features when the observer compares the memory and test stimulus. Even in this case, there can be a systematic loss of some of the frequency components (i.e., high-frequency, small-detail components) during the perception–memory transformation.

Classification image weights and internal noise estimates allow separation between the various schemes regarding how VWM decay could work. In a pure random decay model, we would expect to see an increase in internal noise and a uniform drop in weighting of all features. On the other hand, systematic frequency-based decay should cause a selective decrease in some weights and no increase in internal noise. On the other hand, it is also possible that there could be a more complex form of systematic decay that would not be frequency selective (e.g., change in how symmetries are used). These should change neither the tuning of classification image weights nor internal noise.

### Memory decay tested by using double-pass and ROC methods

The double-pass method (Ahumada, 2002; Burgess & Colborne, 1988) was used in our study to directly test the model of random memory decay (i.e., investigate to what extent forgetting in VWM is due to stimulus-independent internal noise). In the double-pass method, the consistency of the observer's responses in trial pairs where stimuli were identical was measured. Trial pairs were presented in a randomized order within each experimental block. The more consistent the responses between two passes to the identical stimuli (regardless of whether the responses were correct or incorrect), the less they were driven by stimulus-independent internal noise.

The third method in testing VWM decay was receiver operating characteristic (ROC) curves. We found that our somewhat unique design where external noise differs between similar and different trials makes a specific assumption regarding the slopes of the ROC curves if memory decay is driven by internal noise. The ROC curve shows the false alarm and hit rates over all possible decision criteria. The shape of the ROC curve is dependent on the distribution of the so-called decision variable that underlies the observer's decisions in the task. If this distribution is Gaussian, these curves are linear when plotted in standard normal deviate (*z*-score) coordinates. The slope of the ROC curve is determined by the ratio of noise variances between different and similar trials (Green & Swets, 1966; Macmillan & Creelman, 2004).

The random decay model makes a prediction on how the variance of the decision variable should change: The variance of the decision variable is a sum of the stimulus and internal noise variances. Using a standard assumption that internal noise has the same variance in similar and different trials and that the variance of external noise does not change, the ratio of decision variable variances should be above 1 in short retention time but approach 1 with increasing retention time. This is because, in short retention times, the difference in external noise (which is much higher in different trials) should yield a slope that is above 1. Again, if internal noise (which does not differ in similar and different trials) increases with retention time, it becomes more dominant in the variance of a decision variable, and so the ratio of variances approaches unity. On the other hand, if there is no change in internal noise, then the ratio should be independent of retention time. An ROC analysis thus provides a straightforward and complementary way to estimate the effect of internal noise.

## Experiment 1: Forgetting in a grating memory task

Experiment 1 measured VWM for compound gratings where each of 10 grating components was of a randomized phase. Earlier studies employing single gratings in same–different memory tasks have indicated that the memory for SF, for example, is extremely good, with discrimination thresholds being in the hyperacuity range (Magnussen, 2000; Magnussen, 2009; Magnussen & Greenlee, 1999; Magnussen, Greenlee, Asplund, & Dyrnes, 1990; Magnussen et al., 1996). Thresholds are typically not affected by retention time (Magnussen et al., 1990; Magnussen et al., 1996). However, it has been argued that measuring discrimination thresholds for single gratings is not a very sensitive test for VWM, as noise in memory representations due to forgetting must be quite high to affect thresholds markedly (Skottun, 2004). Because our memory stimuli were complex shapes consisting of 10 SF components, the memory task was more demanding and likely to create a high amount of “memory noise.”

### Equipment

The experiments were conducted in a dimly lit laboratory. Stimuli were generated using a Cambridge Research Systems (Rochester, UK) ViSaGe Mark II graphics card with 15-bit luminance resolution, and the stimuli were presented on a Mitsubishi (Tokyo, Japan) Diamond Pro CRT Monitor with a refresh rate of 100 Hz. The resolution of the screen was 800 × 600 pixels with a screen size of 36 × 29 cm. The screen had a maximum luminance of 96 cd/m^2^ and mean luminance of 48 cd/m^2^. The viewing distance (110 cm) was controlled using a chin rest. Experiments were run on MATLAB (MathWorks, Natick, MA) Psychophysics Toolbox extension version 3.0.11 (Brainard, 1997; Kleiner, Brainard, & Pelli, 2007) and custom-built scripts. Results were analyzed using custom MATLAB scripts. JASP 0.13.1.0 was used for the Bayesian ANOVA analyses and R 4.1.1 (R Foundation for Statistical Computing, Vienna, Austria) for the linear mixed modeling. For the linear mixed model analysis, we used the lme4 and lmerTest packages (Bates, Mächler, Bolker, & Walker, 2015; Kuznetsova, Brockhoff, & Christensen, 2017), which use Satterthwaite's method for *P* value estimation (Satterthwaite, 1946).

### Participants

Nine observers (eight women and one man 21–32 years old; mean age, 24.9) with normal or corrected-to-normal vision participated in the study. All observers gave their written consent to participate. Observers were naïve about the purpose of the study; they were only informed that the study concerned VWM. Our procedures were in accordance with the tenets of the Declaration of Helsinki and were accepted by the Research Ethics Committee in the Humanities and Social and Behavioural Sciences of the University of Helsinki.

### Stimuli

The stimuli (Figure 1) were composed of Gabor gratings with 10 SF components (1–10 cycles per degree [cpd]). Each Gabor component had a constant Weber contrast of 12%. The grating phase in each memory stimulus was randomized using a uniform distribution that spanned all phase angles from –180° to 180°. The size of the stimuli was 5° in visual angle (width at half of the maximum Gaussian amplitude).

### Procedure

We used a modified same–different change detection task (Figure 1). Each trial started with a 250-ms fixation screen followed by a 200-ms memory stimulus. After a randomly determined retention time of either 500 ms or 4000 ms (each retention time duration was used in 50% of the trials), the observer was presented with a test stimulus for 500 ms. The observer's task was to indicate, using a button press and rating scale with four options, whether the memory and test stimuli were *definitely similar*, *probably similar*, *probably different*, or *definitely different*. Our task differed from a standard same–different task in that the memory and test stimuli were never completely identical; in similar trials a low level of Gaussian distributed phase noise (σ = 5.7°) was added to the test stimulus, whereas in different trials the noise level was high (σ = 57°). Phase noise values of over ±90° were clipped, preventing angular circularity. Observers started with a short practice session (about 50 trials) to familiarize them with the stimuli and task. Then, 1120 trials per retention time (2240 in total) were measured in blocks of 80 trials in four sessions on separate days (seven blocks per session). For the double-pass response consistency procedure, each trial was presented twice (in randomized order) within a block.

### Data analysis

#### Memory performance

Performance in the memory task was measured using the area under the ROC curve (AUC, *A_z_*), which can be considered a response criterion-free version of probability of correct responses, where 0.5 equals chance level and 1 is perfect performance. We chose to use *A_z_* because it is unambiguous and intuitive and does not require assumption of equal variance for the decision variable, which must be taken into account as the variance of the response variable in different trials was higher than in similar trials. The ROC curves were formed using standard methods by first computing the cumulative hit and false-alarm rates (hit meaning a “similar” response in a similar trial and false alarm a “similar” response in a different trial) for each criterion the observer used for rating scale responses. This was done by computing the cumulative probabilities of responding less than or equal to *o* for each rating scale option *o*, separately for similar and different trials. The ROC curves were then transformed to *z*-scores. On visual inspection, the ROC curves were approximately straight lines, consistent with the assumption that the distribution of the decision variable is Gaussian (Green & Swets, 1966; Macmillan & Creelman, 2004). We then fitted ROC lines to determine slopes *k* and intercepts *b.* It can be shown (Swets & Pickett, 1982) that the area under the ROC curve is given by

(1) $$A_{z}=\Phi \left(\frac{b}{\sqrt{1+k^{2}}}\right)$$

where Φ is the standard cumulative normal distribution function. See the Appendix for linear fits to the ROC curves.

#### Classification images

Let us assume that observers make the “similar” or “different” decisions by comparing the difference between the memory and test stimulus in a set of features. We assume that, due to encoding and memory limitations, these features are internally represented with (possibly) uneven memory strength, represented by internal weights. These weights are described by a template vector **w** whose estimate is the CI. We assume that on a given trial *k* that observers make their decisions using the decision variable *r_s_*_,_*_k_*, which calculates the weighted sum between the template and squared value (as the sign of the phase angle difference is not relevant) of a phase noise vector **n** that was added to the test stimulus:

(2) $$r_{s,k}=w^{T}n_{k}^{2}+r_{i,k}$$

where *r_i_* is random internal noise that is assumed to be independent and Gaussian.

Thus, the larger the phase difference in features that are heavily weighted, the more likely the observer gives a “different” response. We assume further that the observer gives a confidence rating by comparing the real-valued decision variable *r_s_*_,_*_k_* with a set of internal criteria so that the observer gives a rating of *j* when the decision variable falls between criteria *c_j_* and *c_j_*_+1_. Assuming that internal noise is normally distributed, the probability for the observer to give a “different” response with confidence *l* is

(3) $$E\left(r_{t}>c_{l}\right)=\Phi \left(w^{T}n_{k}^{2}-c_{l}\right)$$

where Φ is the standard cumulative normal distribution function.

We used a generalized linear ordinal probit model (GzLM) to estimate the decision weights **ŵ** (i.e., the CI; see Knoblauch & Maloney, 2008). The GzLM is a generalization of the linear regression model where the dependent variable belongs to the exponential family; it uses a nonlinear link function to relate the linear model to the dependent variable and allows the use of a nominal dependent variable with multiple criteria. The model had 10 regressors for the decision weights, three regressors for three internal criteria parameters corresponding to the response categories, and one regressor for the true stimulus category (similar–different). Fitting was done using MATLAB's *mnrfit* function. Finally, as the weights of a CIs are dependent on internal weights, divided by a scalar that is dependent on internal noise (Ahumada, 2002; Knoblauch & Maloney, 2008), we normalized the regressors by dividing the raw weights by their vector length. This allowed us to investigate memory weights independently of internal noise.

Note that the external noise distribution here cannot be assumed to be strictly Gaussian but rather chi-squared, as we assume that the observer compares a sum of squared Gaussian distributed feature values in Equation 2. However, we use the standard approach with Gaussian assumption here, as the chi-squared distribution is approximately Gaussian when the number of features is large. Note also that we could have replaced the square with the absolute value of the phase noise vector, but then we would have been unable to use the Gaussian approximation.

#### Internal noise estimation

We used a generalized version (Kurki & Eckstein, 2014) of the double-pass procedure (Burgess & Colborne, 1988; Green, 1964) that allows the use of rating-scale responses to estimate the ratio of internal to external noise. In this procedure, each trial was presented twice (in randomized order). Assuming that internal responses to both external (feature noise) and internal noise are approximately Gaussian, it is possible to estimate their relative amounts, given the agreement between two passes and response criteria. More specifically, the internal response to a stimulus is dependent on the response to external noise (*r_e_*) and on internal noise (*r_i_*) so that *r_s_* = *r_e_* + *r_i_*. For simplicity, we can assume that the standard deviation of the model's response to external noise is 1 and internal noise standard deviation is σ*_i_*. We let ***c*** = [–∞, *c*_1_, *c*_2_, *c*_3_] be the response criteria for four confidence levels and then estimated the ratio of internal to external noise standard deviations, which is the same as σ*_i_*. For two passes of the same stimuli, *r_e_*_1_ = *r_e_*_2_ = *r_e_*. The probability of response pair *a*_1_ = *l* and *a*_2_ = *k* is

(4) $$\begin{array}{ccc} & & p\left(a_{1}=l,\;a_{2}=k|r_{s}\right)=p\left(c_{l-1}<r_{e}+r_{i,1}<c_{l}\right)\\ & & \;p(c_{k-1}<r_{e}+r_{i,2}<c_{k}) \end{array}$$

(5) $$\begin{array}{ccc} & & =\left(\Phi \left(\frac{c_{k}-r_{e}}{\sigma_{i}}\right)-\Phi \left(\frac{c_{k-1}-r_{e}}{\sigma_{i}}\right)\right)\\ & & \;\left(\Phi \left(\frac{c_{l}-r_{e}}{\sigma_{i}}\right)-\Phi \left(\frac{c_{l-1}-r_{e}}{\sigma_{i}}\right)\right)\; \end{array}$$

where Φ(*x*) is the standard cumulative normal distribution. The expectation for the consistency can then be solved by integrating over all external noise values (for details, see Kurki & Eckstein, 2014). As noted previously, strictly speaking the probability density function for *r_e_* is approximately chi-squared with degrees of freedom *k*, depending on how many features are weighted. However, as classification images show that humans use multiple features, and they do not show a difference in the number of features in short and long duration, we simply approximate the distribution of *r_e_* by a Gaussian distribution.

Only different trials were analyzed, as we found that we could not reliably estimate internal noise in similar trials. It is probable that performance in similar trials is overwhelmingly limited by internal noise, as external noise was only 1/10 of the amount of noise in different trials, making the internal-to-external noise ratio an unstable measure.

#### ROC analysis

We analyzed the slopes of ROC curves for the short and long retention times using combined data from both experiments, using the same methods as for the performance analysis. As this study was originally designed for CI and double-pass analyses, we wanted to increase the statistical power of ROC curve estimation by combining the data from both experiments. Furthermore, we expected that memory for spatial and radial frequencies would behave similarly, so there was no theoretical reason why data could not be combined. However, we wanted to take the possible differences in the experiments into account in our analysis. Thus, we used linear mixed modeling with experiment, retention time, and their interaction as fixed effects and observer as the random effect in our statistical analysis of ROC slopes.

The slope of the ROC curve *k* is determined by *k* = σ*_d_*/σ*_s_*, the ratio of decision variable standard deviation in different and similar trials (Green & Swets, 1966; Macmillan & Creelman, 2004). Note that similar trials are the “signal” distribution of the SDT model, and different trials that had 10 times more phase noise are the “noise” distribution. Thus, we assume that the variance of the decision variable for the different trials will be greater than in the same trials and *k* to be >1. When inspecting the slopes, note that in many ROC studies the “signal” trials have had more variance than the “noise” trials; thus, *k* < 1. Also, in recognition memory studies, the signal is typically defined as old trials, where there is more variance resulting in *k* values less than 1.

Assuming that external and internal noise are independent, the slope can be written as a square of the sum of stimulus-dependent external noise and stimulus-independent internal noise standard deviation:

(6) $$k=\frac{\sigma_{d}}{\sigma_{s}}=\sqrt{\sigma_{ed}^{2}+\sigma_{it}^{2}}/\sqrt{\sigma_{es}^{2}+\sigma_{it}^{2}}\;$$

where σ^2^*_ed_* and σ^2^*_es_* are external noise variance in the different and similar trials, and σ^2^*_it_* is the internal noise variance at retention time *t*, assumed to be the same in similar and different trials.

In the random decay model, memory decay is driven by internal noise and σ^2^*_it_* grows with retention time, but σ^2^*_ed_* and σ^2^*_es_* remain the same, and *k* approaches 1. On the other hand, if internal noise does not change with retention time, there should be no change in *k* over short durations. We estimated *k* for each retention time using the standard method with least-squares linear regression.

Because we have a double-pass estimate for the internal noise levels σ^2^*_it_*, it would, in principle, also be possible to directly test the random decay model by predicting the ROC slopes using Equation 5, as all terms could be estimated from double-pass data. However, as noted before, the external noise-to-internal noise ratio could not be reliably estimated in similar trials, and the external noise standard deviation was 10 times higher in different trials. We therefore approximated the prediction of the model by using an approximation where external noise has negligible (zero) contribution to responses in similar trials by setting the parameter σ^2^*_es_* to 0. That is, performance would be completely limited by internal noise. This enabled us to predict the ROC curve slopes and compare them with observed values.

### Results

#### Memory performance

**Figure 2.**
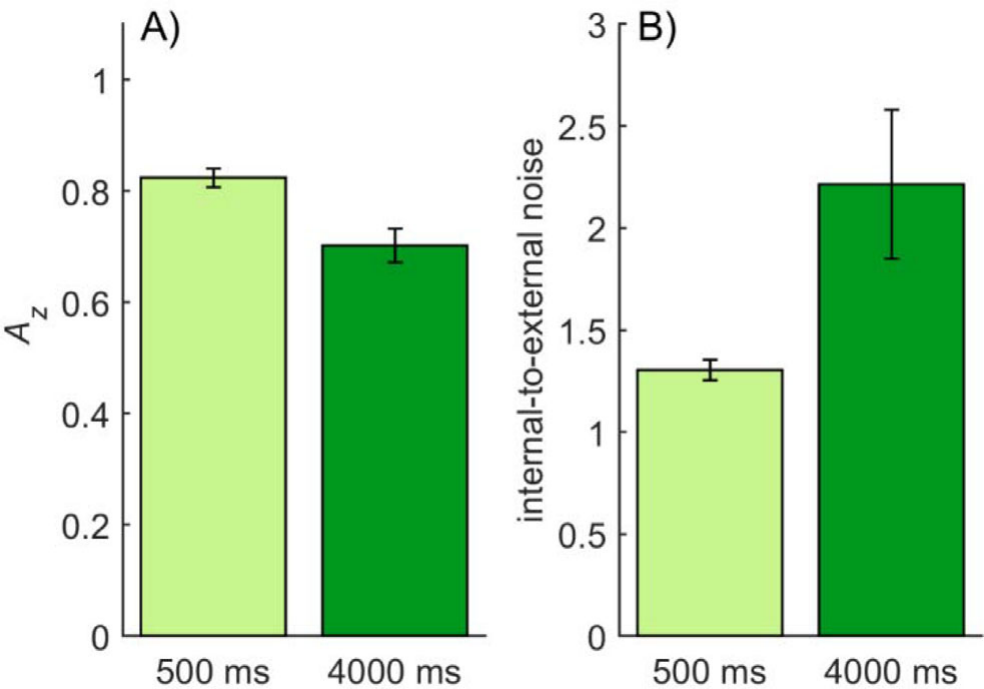
(A) Mean memory performance (*A_z_*) indicating how well observers could discriminate between similar and different trials in Experiment 1. (B) The mean internal noise level measured by the double-pass response consistency method and expressed as the ratio of internal-to-external noise in Experiment 1. Error bars in both figures represent the standard error of mean (*SEM*).

Observers showed substantial forgetting when they tried to memorize the spatial structure of compound gratings (Figure 2A): memory performance (*A_z_*) decreased about 15% when compared between retention times of 500 ms and 4000 ms (*M*_500_ = 0.82, *SD*_500_ = 0.05; *M*_4000_ = 0.70, *SD*_4000_ = 0.08), with *t*(8) = 7.18, *p* < 0.001, and *d* = 1.78. Measured as *d*′*_e_* (Green & Swets, 1966; Macmillan & Creelman, 2004), a version of *d*′ adapted to situations where response variables in the signal and distractor have different variances as was the case here, performance dropped approximately 42% with the longer retention time (*d*′*_e_*_500_ = 1.50; *d*′*_e_*_4000_ = 0.89), and the difference between retention times was statistically significant in a repeated measures *t*-test, where *t*(8) = 11.08, *p* < 0.001, and *d* = 2.10. Because all trials were presented twice for the double-pass method, we also compared performance in the first and second passes of the trials. There was no difference in *A_z_* in the first pass (*M*_1_ = 0.77, *SD*_1_ = 0.06) or second pass (*M*_2_ = 0.77, *SD*_2_ = 0.05), with *t*(8) = 0.09, *p* = 0.927, and *d* = 0.03.

#### Classification images

**Figure 3.**
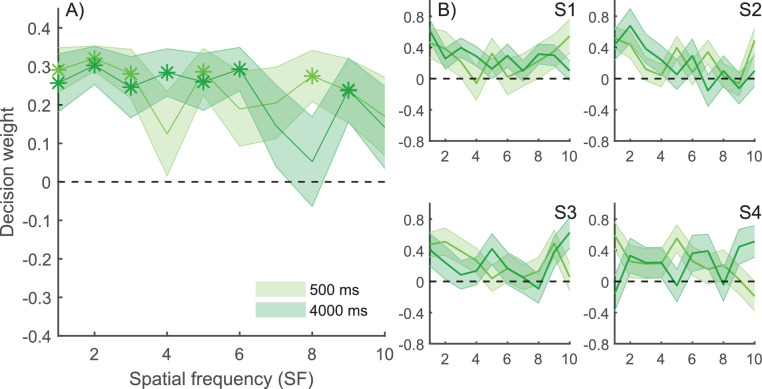
CIs in Experiment 1. (A) Average normalized CI decision weights (arbitrary units) for short (500 ms) and long (4000 ms) retention times. High decision weights indicate that phase noise in those SFs affected observers’ memory-based responses more; a weight of zero indicates no effect. Although almost all SF components had weights larger than zero, the weights in low SFs were slightly higher. The weights that are larger than zero at a 5% significance level are marked with asterisks (*). There were no statistically significant differences in memory tuning between the two retention times. (B) CI weights for four representative observers. Shaded areas represent the *SEM*.

The average CIs showed rather uniform weights across all SFs, with higher frequencies having marginally less weighting (Figure 3A). We tested whether average normalized CI weights for each SF and retention time were non-zero using a family of one-sample *t*-tests where the *p* values were false discovery rate (FDR) corrected for multiple comparisons (Benjamini & Hochberg, 1995). Seven out of 10 SF components at the 500-ms and five out of 10 components at the 4000-ms retention time had *p* values smaller than 0.05 (indicated by asterisks in Figure 3A). This result implies that the VWM for compound gratings can maintain a broad range of SF components. Retention time did not change weighting in any apparent fashion; both retention times showed similar weights. Figure 3B shows CI weights for four representative observers. Although there was greater variation compared to the average results, slightly larger weights were concentrated in lower frequencies.

The differences between the normalized CI weights at two retention times and different SF components was tested using a two-way repeated-measures ANOVA. No effects of retention time, SF, or their interaction were found: For retention time, *F*(1,8) = 0.14, *p* = 0.714, and η*_p_*^2^ = 0.018; for SF, *F*(1,8) = 1.02, *p* = 0.431, and η*_p_*^2^ = 0.113; and for interaction, *F*(1,8) = 0.68, *p* = 0.721, and η*_p_*^2^ = 0.079. The lack of main effect for SF shows that there was no evidence of differences in the weighting of various SF components; that is, no evidence that forgetting in VWM resulted from a change in memory tuning. The lack of a main effect for retention time indicates that there is no evidence of overall magnitude of weights changing with time, which was expected, because of normalization. The lack of interaction suggests that there is no evidence of weighting changing with retention time.

We also conducted a Bayesian two-way repeated-measures ANOVA for the average normalized CI weights in the two retention times and SFs. The inclusion Bayes factor, which indicates the level of evidence for including a factor, was 0.115 for retention time, 0.031 for SF, and 0.003 for their interaction. These results support the observation that neither retention time nor SF had an effect on normalized CI weights.

#### Internal noise

Figure 2B shows the average internal-to-external noise ratios for the two retention times, as measured by the double-pass method. The level of internal noise increased about 70% between memory times of 500 ms and 4000 ms (*M*_500_ = 1.30, *SD*_500_ = 0.15; *M*_4000_ = 2.21, *SD*_4000_ = 1.10), where *t*(8) = 2.36, *p* = 0.046, and *d* = 1.16. In a one-way paired-samples Bayesian *t*-test, where the alternative hypothesis was that internal noise level increased at the 4000-ms retention time, the Bayes factor was 8.144 in favor of the alternative hypothesis. This result provides evidence for the idea that internal noise increases over time and causes forgetting.

## Experiment 2: Forgetting in a shape memory task

**Figure 4.**
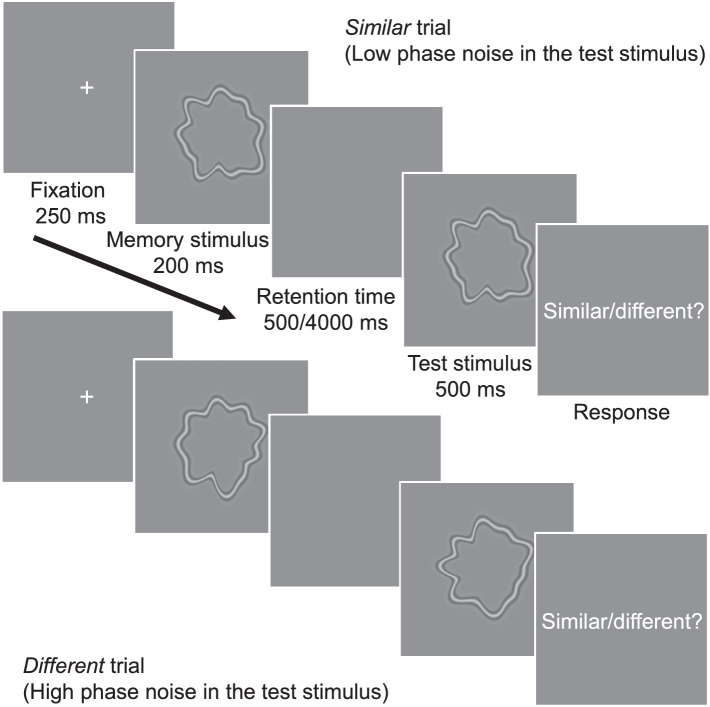
Procedure in Experiment 2. The procedure was identical to Experiment 1 except for the stimuli, which were circular RF shapes.

Experiment 2 tested VWM for complex shapes of RF patterns (Bell & Badcock, 2008; Bell & Badcock, 2009; Loffler, 2015; Wilkinson et al., 1998). RF patterns are formed by modulating the radius of a circle with sinusoidal functions of a polar angle (Figure 4). RF patterns can represent many real-world shapes while providing a good control for low-level stimulus visibility (Loffler, 2008; Loffler, 2015). Therefore, they have been widely used to investigate mid-level visual mechanisms underlying contour and shape integration and also VWM (Salmela, Mäkelä, & Saarinen, 2010). Even though RF patterns have many qualities similar to the shapes of real objects, they cannot be easily verbalized, thus providing a link between low- and high-level vision. In Experiment 2, we measured the VWM for RF patterns composed of 10 RF components.

### Equipment

The equipment was identical to that in Experiment 1.

### Participants

Nine new observers (seven women and two men, 21–37 years old; mean age, 26.22) participated in Experiment 2. All observers had normal or corrected-to-normal vision and gave their written consent to participate in the study. Observers were informed that they were participating in a study on VWM but were naïve about the exact purpose of the experiment.

### Stimuli

The stimuli were circular contours (mean radius 1.8° in visual angle) that were modulated by 10 sinusoidal RF components (1–10 cpd) with a constant amplitude, randomized phase, and SF of 3.8 cpd (Figure 4). All 10 components had a Weber contrast of 5%.

### Procedure

The procedure was identical to Experiment 1, except that the stimuli were circular shapes with varying RF phase. After a short practice session (about 50 trials), observers performed 1120 trials per retention time (2240 in total) in blocks of 80 trials, in four sessions held on separate days (seven blocks per session). As in Experiment 1, each trial was repeated two times in a random order inside a block for double-pass analysis.

### Data analysis

The procedures used to analyze data were identical to those in Experiment 1.

### Results

#### Memory performance

**Figure 5.**
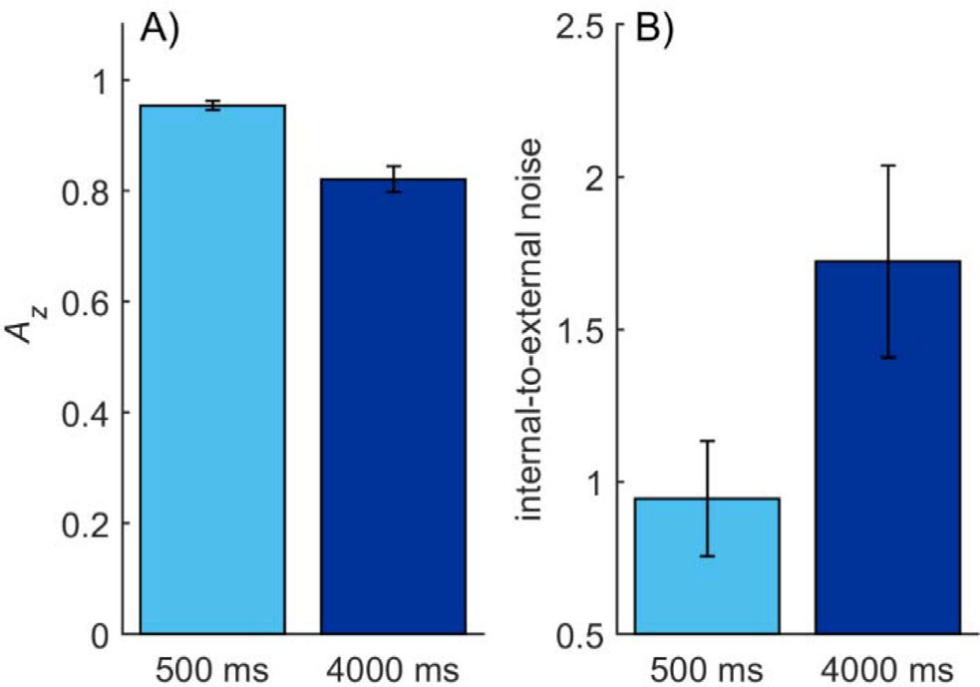
(A) Average memory performance (*A_z_*) in Experiment 2 with RF patterns. (B) Average ratio of internal to external noise as measured by the double-pass response consistency method in Experiment 2. Error bars in both figures represent the *SEM*.

Performance (*A_z_*) in the memory task dropped approximately 14% between the 500-ms (*M* = 0.95, *SD* = 0.02) and 4000-ms (*M* = 0.82, *SD* = 0.07) retention times, where *t*(8) = 6.18, *p* < 0.001, and *d* = 2.70 (Figure 5A). For *d*′*_e_*, the difference in performance between retention times was about 45% (*d*′*_e_*_500_ = 2.58; *d*′*_e_*_4000_ = 1.41), and it was statistically significant in a repeated-measures *t*-test: *t*(8) = 8.67, *p* < 0.001, and *d* = 3.17. There was no difference in performance in the first (*M*_1_ = 0.90, *SD*_1_ = 0.03) and second (*M*_2_ = 0.90, *SD*_2_ = 0.03) passes of the trials: *t*(8) = 1.70, *p* = 0.128, and *d* = 0.57.

#### Classification images

**Figure 6.**
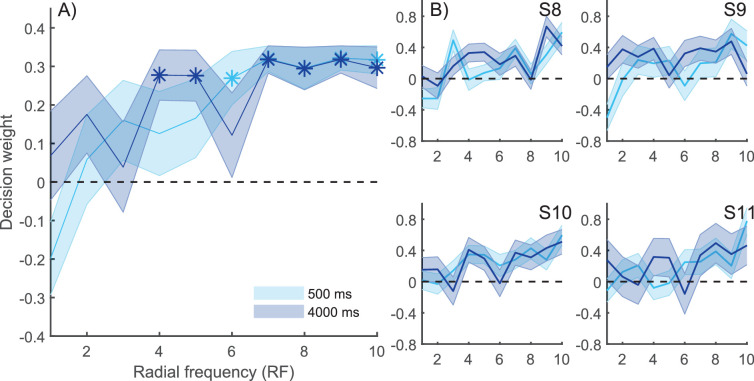
CIs in Experiment 2. (A) Average CI weights for short (500 ms) and long (4000 ms) retention times. High memory decision weights mean that those RF components affected the observer's memory-based responses more; a zero weight means that the RF component had no effect on observers’ responses. CIs had more weighting in high RFs, implying that VWM for shape is supported by high RFs. The weights larger than zero at a 5% significance level (corrected for multiple comparisons using FDR) are marked with asterisks (*). Importantly, there were no differences in the memory tuning between short and long retention times. (B) Individual CIs for four representative observers. Shaded areas represent the *SEM*.

The average CIs in Experiment 2 (Figure 6A) show that several RF components were weighted for memory-based decisions. However, unlike in Experiment 1 on compound gratings, there were now clear signs of memory tuning, as high RFs were weighted more than low frequencies. The individual CIs of four representative observers show a similar pattern of tuning (Figure 6B). We confirmed whether normalized CI weights differed from zero in each RF and at the two retention times using multiple one-sample *t*-tests with FDR correction for multiple comparisons. Five out of 10 RF components at the 500-ms and six out of 10 RF components at the 4000-ms retention times had memory weights significantly different from zero (*p* < 0.05) (Figure 6A). As in Experiment 1, the CIs appeared to have similar shapes at short and long retention times.

A two-way repeated-measures ANOVA on normalized CI weights showed no significant main effect for retention time, with *F*(1,8) = 0.44, *p* = 0.525, and η*_p_*^2^ = 0.052; however, the main effect for the RF component was statistically significant, with *F*(1,8) = 4.44, *p* < 0.001, and η*_p_*^2^ = 0.357. Thus, there was a memory tuning in RF component weights. Critically, the interaction between retention time and RF components was not significant, with *F*(1,8) = 1.47, *p* = 0.177, and η*_p_*^2^ = 0.155), implying that there is no evidence of RF components decaying selectively at the 4000-ms retention time. We confirmed this observation with a two-way Bayesian repeated-measures ANOVA; the inclusion Bayes factor was 1076.210 for radial frequency, indicating that there was a strong effect for RF. The inclusion Bayes factors for retention time and interaction of retention time and RF were 0.204 and 0.202, respectively. Based on this analysis, there was no effect of retention time or the interaction of retention time and RF on CI weighting, indicating that forgetting is not dependent on the RF of the shape components.

#### Internal noise

The ratio of internal to external noise increased about 83% from the 500-ms (*M* = 0.94, *SD* = 0.57) to the 4000-ms (*M* = 1.72, *SD* = 0.93) retention time (Figure 5B). The increase was statistically significant, with paired samples *t*-test *t*(8) = 2.47, *p* = 0.038, and *d* = 1.00. According to a one-tailed Bayesian paired-sample *t*-test, the Bayes factor was 4.328, suggesting that the alternative hypothesis was more likely to hold than the null hypothesis. This favors the idea that internal noise level was affected by retention time.

### ROC-analysis

**Figure 7.**
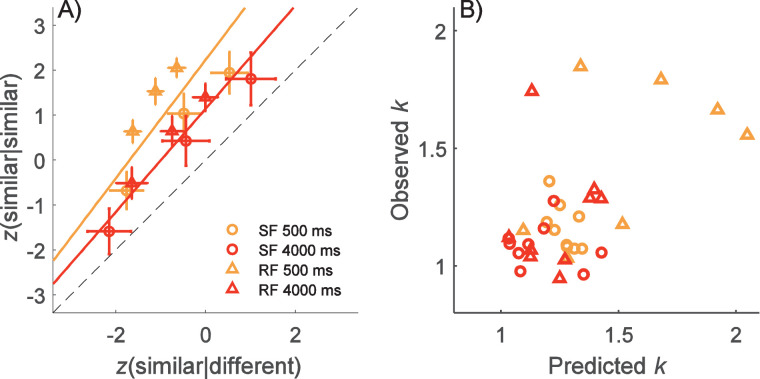
ROC analysis. (A) The *z*-score–transformed ROC curves representing hit (similar responses when stimuli were similar) and false alarm (similar response when stimuli were different) probabilities of confidence ratings. Each point is an average over nine observers, and error bars represent the *SEM*. Lines show mean ROC curves over all observers for both retention times and experiments. The diagonal is plotted with a dashed line. (B) Comparison of the observed ROC curve slopes (observed *k*) to the prediction of random decay model (predicted *k*) using Equation 5 where the level of internal noise is estimated from the double-pass prediction.

The average points of ROC curves over all observers in both experiments and for both retention times are depicted in Figure 7A, which shows the hit and false alarm probabilities for various criteria as *z*-scores (see the individual ROCs in the Appendix). The mean slope for the observers’ ROC curves for the 500-ms retention time (across experiments) was 1.32 (*SD* = 0.26), which differed significantly from 1, with *t*(17) = 5.17, *p* < 0.001, and *d* = 1.23. The slope for the ROC curve for the 4000-ms retention time was shallower: *M* = 1.15 (*SD* = 0.19). The average intercepts of the model were 2.26 (*SD* = 0.93) for the 500-ms retention time and 1.17 (*SD* = 0.60) for the 4000-ms retention time. We ran a linear mixed model analysis with experiment, retention time, and their interaction as fixed effects and observer as a random effect to compare ROC slopes in different conditions. We found an effect in ROC slopes for both fixed effects: For retention time, β = –0.26, *t*(17) = –3.68, and *p* = 0.002; for experiment, β = –0.30, *t*(26.95) = –3.19, and *p* = 0.004. There was no effect on the interaction of retention time and experiment, with β = 0.19, *t*(16) = 1.84, and *p* = 0.085, indicating that there was no evidence of ROC slopes behaving differently in Experiments 1 and 2. The effect on the intercept was statistically significant, with β = 1.47, *t*(26.95) = 21.94, and *p* < 0.001, reflecting the fact that performance declined with the longer retention time.

Figure 7B shows the prediction of the ROC slopes for the random decay model from the double-pass internal noise estimates, with the additional assumption that external noise in the similar condition (σ*_es_*) is 0. Predicted slopes were 1.48 for the 500-ms retention time and 1.20 for the 4000-ms retention time. We found that the prediction was reasonably good, with relatively high correlation between observers’ predicted and observed slopes (ρ = 0.557; *p* < 0.001). In Figure 7B, there seems to be five outliers with an observed slope greater than 1.5; to check, we calculated correlation without these outliers. We found that correlation between observed and predicted slopes remained high (ρ = 0.531; *p* = 0.003). We also checked that the correlation could not be explained merely by performance (*A_z_*) in the memory task. We found that the partial correlation controlling for *A_z_* was lower, but it remained statistically significant (ρ = 0.386; *p* = 0.039).

## Discussion

We investigated the decay of visual features in VWM using CIs, internal noise estimation, and ROC analysis. Two models of memory decay were tested: (1) systematic changes in memory representations in the course of forgetting, and (2) random corruption of representations due to internal noise when all features decayed uniformly. In terms of systematic decay, we tested a model where some frequency components would systematically decay first. Both compound gratings and RF patterns were used in our same-different memory task to probe VWM at different stages of visual processing.

### Memory for spatial frequency

CIs showed that several SF components were weighted systematically (i.e., had significantly positive weights) for memory-based decisions, indicating that VWM representations contain several SF components. Interestingly, even though the observers showed significant forgetting in the memory task, there were no signs of systematic changes in memory weighting of SF components at the long retention time as predicted by Gold et al. (2005). This strongly suggests that decay in VWM is not specific to certain SF components, but instead all SF components seem to deteriorate equally.

Studies that have measured sensitivity for SF using detection thresholds have typically found bandpass tuning that peaks at around 2 to 4 cpd (Campbell & Robson, 1968; De Valois & De Valois, 1980). We did not observe any systematic SF tuning in VWM, but all SF components were weighted with rather uniform weights. This result was not unexpected, as we used suprathreshold compound gratings, which have relatively uniform sensitivity/contrast across SFs (Georgeson & Sullivan, 1975).

Unlike the results from studies on single gratings (Magnussen et al., 1990; Magnussen et al., 1996), we found a clear effect for retention time on memory performance. Our result suggests that memory for SF may not be as perfect as earlier thought. Skottun (2004) argued that internal noise (“memory noise”) has only a moderate effect on the discrimination thresholds of simple gratings, and the increase in internal noise must be considerable to reveal any forgetting effects. By using compound gratings with complex structures, the internal memory noise increased at longer retention times, which might have enabled our task to be sensitive enough to show the effects of retention time on memory performance. Notably, we were able to measure SF tuning for VWM using CIs, as CIs have previously been used only in perceptual tasks.

### Memory for shape radial frequency

The CIs for shape memory in Experiment 2 revealed that several RF components were weighted for memory-based decisions. In line with the results of Experiment 1, the weighting of RF components did not, however, show any systematic changes with retention time, even when observers’ performance in our modified same–different task was significantly poorer at the long retention time. This result thus suggests that VWM decay was not specific to certain shape features, but forgetting affected all features equally. Unlike in Experiment 1 on compound gratings, there was a clear “tuning” of RF components, so that higher RF components had relatively larger weights than lower ones. This implies that higher RF components are more efficiently used in the similar–different task. The tuning is in line with the sensitivity measurements obtained in shape perception experiments (see, for example, Wilkinson et al., 1998). Better memory for higher RF components might thus be explained by more efficient perceptual encoding.

### Internal noise and memory decay

We found that internal noise, as estimated using double-pass response consistency, increased with retention time. The noise increase was substantial in both Experiments 1 and 2, on average 77%. The increase implies that forgetting in VWM could be due to internal noise accumulation. Moreover, ROC curves were approximately linear when plotted in *z*-scores. The slope of the ROC curves, which reflects the ratio of combined external and internal noise in different versus similar trials, decreased with retention time. As external noise exhibited much greater variance in the different trials, we expected this ratio to be well above 1, as was found for short retention time. A decrease of the slope closer to 1 gives further evidence to the idea that performance is limited by an increase in stimulus-independent noise. As we consistently found that the ROC slopes in long and short retention times differed, our analysis supports a model where forgetting is caused by an increase in internal noise. Moreover, we found reasonably good agreement in slopes when estimated from the double-pass and ROC data. This further suggests that a straightforward increase in internal noise with retention time could explain the findings without any extra assumptions. On the other hand, it is not easy to explain these findings through systematic forgetting with no change in internal noise.

It must be noted that comparing ROC curves makes some assumptions that are currently debated in VWM literature. Here, we assume that ROC slope can be described as the ratio of decision variable standard deviation in different and similar trials (see Equation 5), which is an assumption embedded in signal detection theory (Green & Swets, 1966; Macmillan & Creelman, 2004). However, some models of VWM reject this idea, such as the high-threshold (HT) models of VWM (for a review, see Yonelinas & Parks, 2007). Yonelinas and Parks (2007) stated that HT models predict nonlinear *z*-transformed ROC curves (see evidence for the HT model in Rouder, Morey, Cowan, Zwilling, Morey, & Pratte, 2008), a finding that contradicts our observation of linear *z*-transformed ROC curves. Thus, our results do support a model where there is at least a signal detection component in VWM, but we cannot rule out the possibility that our assumptions do not hold. Then again, we did find increasing internal noise based on the double-pass analysis, which does not make assumptions about the nature of VWM.

### Previous studies on VWM decay

Harvey (1986) studied VWM for compound gratings using a same–different memory task and regression-like technique to estimate the retention of each SF component. His results showed that memory tuning did not change as a function of retention time, although observers’ memory performance clearly decreased at longer retention times, in accordance with our results. Gao and Bentin (2011) tested how various SFs decay in VWM using face stimuli composed of either low or high broadband SFs. The observers had to first memorize a set of faces and then make subsequent old/new decisions between two test faces. Gao and Bentin (2011) found that memory representations of faces in both frequency bands decayed at a similar rate, suggesting there are no systematic differences in memory decay between different SFs. It must be noted, however, that Gao and Bentin did not directly probe whether or not internal noise affected forgetting in VWM.

Although our results were in line with those of studies by Harvey (1986) and Gao and Bentin (2011), Gold et al*.* (2005) reported conflicting results. In their experiments on VWM, internal noise level as measured by the double-pass method did not change with retention time; instead, sampling efficiency was found to decrease. Even though not directly tested, the effect on sampling efficiency implies that some spatial frequencies could undergo more decay than others. Because the task of Gold et al*.* (2005) was similar to ours, it is not clear why we did not find any systematic decay of certain frequencies and instead observed an increase in internal noise levels. Although more experimentation will be needed to clarify the reason for the discrepancy between the results of Gold et al*.* (2005) and ours, one explanation might be the very different stimuli used in these two studies. Gold et al*.* (2005) employed textures with bandpass random noise, whereas our memory stimuli were compound gratings and RF patterns. Textures could perhaps be particularly difficult to encode in visually retrievable form and could promote verbal encoding strategies (e.g., “large blob in the upper left texture corner”).

In VWM literature there has been different accounts on whether VWM representations are encoded as complete objects or a collection of features. Zhang and Luck (2009) found that objects suddenly dropped from memory, supporting the object-based view. Fougnie and Alvarez (2011), however, found that memory errors occurred independently from different features of an object, and they suggested that features are encoded as separate units in VWM. On the other hand, we observed uniform decay in all frequencies, finding no evidence for memory for frequencies failing independently. Although this study cannot conclusively answer whether VWM representations are object or feature based, CIs could be used in future research to study this question.

## Conclusions

A novel variant of the CI method and other SDT-based methods was used to investigate the mechanisms of VWM decay. CIs provide a powerful tool that can reveal what features are retained in VWM for various retention times. Our results strongly support the idea that memory decay in VWM does not result from the systematic forgetting of visual features but rather from a uniform increase in random internal noise as time passes.

## Figures and Tables

**Figure 8.**
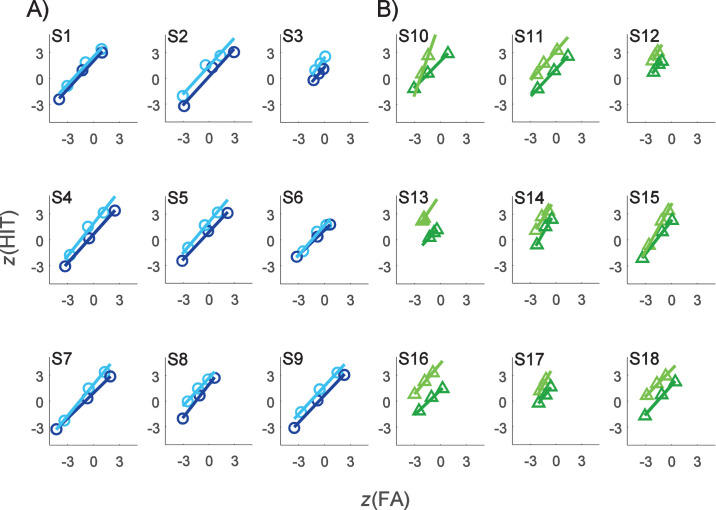
The *z*-transformed ROC curves plotted separately for each observer representing hit and false alarm probabilities of confidence ratings. Lines show the best fitting least-squares ROC curves for each observer for both retention times. (A) The *z*-transformed ROCs for observers (Obs) in Experiment 1, where the stimuli were compound gratings. (B) The *z*-transformed ROCs for observers in Experiment 2, where the stimuli were radial frequency patterns.

## Appendix

### Individual *z*-transformed ROC curves

Figures 8A and 8B show *z*-transformed ROC curves for all observers in Experiments 1 and 2, respectively. By visual inspection, no apparent nonlinearity can be seen in the *z*-transformed ROC curves. Further, we found that the linear model provided very good fits for all observers, as *R*^2^ values in Experiment 1 were on average 0.986 (range, 0.968–0.998; *SD* = 0.011) and 0.994 (range, 0.970–1.000; *SD* = 0.009) for the 500-ms and 4000-ms retention times, respectively. The corresponding values in Experiment 2 were 0.981 (range, 0.937–0.999; *SD* = 0.025) and 0.993 (range, 0.978–0.999; *SD* = 0.007).
